# *Chrysanthemum coronarium* L. Protects against Premature Senescence in Human Endothelial Cells

**DOI:** 10.3390/cimb44120397

**Published:** 2022-11-23

**Authors:** Mi Jeong Sung, Ae Sin Lee

**Affiliations:** Research Group of Aging and Metabolism, Food Functionality Research, Korea Food Research Institute, Jeonju 55365, Jeollabuk-do, Republic of Korea

**Keywords:** *Chrysanthemum coronarium L.*, human umbilical vein endothelial cells, senescence, sirtuin 1, endothelial nitric oxide synthase

## Abstract

The senescence of vascular endothelial cells (EC) leads to vascular dysfunction. However, the molecular mechanisms of EC senescence and its associated pathophysiological changes have not yet been clearly studied. This study sought to inspect the *Chrysanthemum coronarium* L. (CC) extract’s mechanism in preventing premature senescence of EC. A senescent endothelial cell model was created in human umbilical vein endothelial cells (HUVECs) with 100 µmol/L H_2_O_2_ treatment for 24 h. The effect of CC on senescent HUVECs was elucidated by measuring the activity of β-galactosidase (SA-β-gal), which exhibits an aging-related phenotype. SA-β-gal activity increased to 13.2 ± 2.85% in H_2_O_2_-treated HUVECs, whereas this activity was attenuated in the CC group. Immunoblot analyses revealed that p21, p53, and PAI-1 levels increased in the senescent HUVECs; however, the levels decreased in the HUVECs treated with various concentrations of CC (10, 20, and 50 μg/mL). The CC extract reduced the production of reactive oxygen species and reversed the decrease in NO production. Additionally, pretreatment with an Nω-nitro-l-arginine methyl ester (eNOS inhibitor) and nicotinamide (sirtuin 1 inhibitor) inhibited the anti-senescent effect of CC extract in HUVECs. Taken together, this study validated the novel endothelial protective effect of CC extract and its prevention of senescence in HUVECs through the mechanism regulated by eNOS and SIRT1 expression.

## 1. Introduction

Aged cells secrete inflammatory cytokines, which can be induced by various stimuli such as oxidative stress and persistent inflammation, to gradually arrest the cell cycle. It has been established that cell cycle arrest is mediated by two cyclin-dependent kinase inhibitors, p21, and p16, and sustained DNA damage signaling induces a senescent cell phenotype [1,2,3].

The senescence of vascular endothelial cells (EC) leads to vascular dysfunction. Senescent ECs are characterized by endothelial nitric oxide (NO) production, DNA damage, elevated vascular inflammation, and dysregulation of the cell cycle. Aged ECs appear flat and expanded, and all features associated with cellular senescence become increasingly evident, including pluripotent nuclei [4]. Aging of ECs results in vascular structural and functional changes that advance thrombosis, inflammation, and atherosclerosis through the development of cardiovascular disease and angiogenesis, and vascular integrity [5]. The molecular mechanisms of EC senescence and the associated pathophysiological changes are not yet fully understood.

*Chrysanthemum coronarium* L. (CC), called Glebionis Coronaria, is a flowering plant species belonging to the Asteraceae family. Notably, CC is rich in beta-carotene, iron, potassium, calcium, dietary fiber, and various physiologically active substances [6].

Endothelial dysfunction is marked by impaired nitric oxide (NO) bioavailability derived from endothelial nitric oxide synthase (eNOS), which signals the onset of atherosclerosis. Activated endothelial NO can prevent oxidative stress in ECs by promoting a subsequent production process that delays senescence in ECs [7,8]. In ECs, Sirtuin 1 (SIRT1) and eNOS synergistically regulate each other. The SIRT1/eNOS axis is an insightful point in vascular senescence. Additionally, SIRT1 present in ECs plays a vasoprotective role in sustaining endothelial function by controlling various substrates, including liver kinase B1 and forkhead box O1 [9,10]. Mutual regulation between SIRT1- and eNOS- related signaling pathways has also been reported to promote endothelial functions [11].

The main purpose of this study was to confirm the inhibitory effect of CC extract on oxidative stress-associated cell aging, as well as the possibility of regulating NO and SIRT1 to achieve vasoprotective effects. Therefore, in this study, we sought to explore the mechanism related to CC extract’s inhibitory effect on endothelial cell senescence.

## 2. Materials and Methods

### 2.1. Preparation and Analysis of Chrysanthemum coronarium L. (CC) Extract

For the CC extract used in the experiment, fresh CC was purchased in the market of South Korea, freeze-dried, and powdered. Powdered CC was suspended in 70% ethanol in a ratio of 1:4 (*w*/*v*) and then extracted at room temperature overnight. This solution was filtered, concentrated, and freeze-dried at −80 °C, and the remainder was dissolved in DMSO used as the CC extract. Major compounds of the *Chrysanthemum coronarium* L. extract were analyzed using ultra-performance liquid chromatography-quadrupole time-of-flight mass spectrometry (UPLC-Q-TOF MS) (Waters, Milford, MA, USA). For this procedure, the extract was injected into an Acquity BEH C18 column (2.1 × 100 mm, 1.7 μm, Waters) equilibrated with mobile phase A (0.1% formic acid in water) and eluted using a linear gradient with mobile phase B (acetonitrile containing 0.1% formic acid). The eluted compounds ionized by negative electrospray ionization (ESI) were detected using Q-TOF MS under the following conditions: capillary voltage of 2 kV, sampling cone voltage of 40 V, desolvation temperature of 400 °C, source temperature of 100 °C, a scan range of 50–1500 *m*/*z*. Leucine-enkephalin ([M + H] = 556.2771) was used as lock mass, and the MS/MS data were collected using collision energy ramps of 10–40 eV. Compounds were tentatively identified using the online databases connected to UNIFI software (Waters).

### 2.2. Cell Culture and Materials

For the ECs, primary human umbilical vein endothelial cells (HUVECs) were provided by Lonza (Walkersville, MD, USA). EBM^TM^-2 Basal Medium supplemented with SingleQuots^TM^ supplements, including hFGF-B, VEGF, R3-IGF-1, and heparin, was used to culture the cells at 37 °C and 5% CO_2_. HUVECs were treated with 100 μM H_2_O_2_ for 24 h to stimulate cellular senescence, and CC was added in different concentrations (10, 20, and 50 μM).

### 2.3. Western Blot Analysis

Western blotting was conducted to analyze protein expression as previously described [12]. To obtain proteins, RIPA buffer containing protease and phosphatase inhibitor was added to the adherent cells, and the protein fraction was obtained via centrifugation following physical destruction. 20 µg of protein samples were mixed with 5 × sample buffer, boiled at 95 °C for 7 min, separated by sodium dodecyl sulfate-polyacrylamide gel electrophoresis, and then transferred onto a 0.2 µm PVDF membrane. After protein transfer, the PVDF membrane was blocked with a blocking buffer containing 5% skim milk and 1.5% BSA for 1 h, treated with a primary antibody (1:1000) in TBST at 4 °C, followed by the addition of an HRP-conjugated secondary antibody for chemiluminescence detection. The primary antibodies used were anti-p21 (#2947), anti-p53 (#2524), anti-PAI-1 (#49536) (Cell Signaling Technology, Beverly, MA, USA), anti-p-ERK (sc-7383), anti-ERK (sc-514302), anti-Sirt1 (sc-15404), anti-β-actin (sc-47778) (Santa Cruz Biotechnology, Santa Cruz, CA, USA), and anti-eNOS (ab76198) (Abcam, Cambridge, UK). ECL detection reagent (Amersham ECL; GE Healthcare, Pittsburgh, PA, USA) was used for detection, and the blot was analyzed using the ChemiDoc XRS Imaging System (Bio-Rad Laboratories, Hercules, CA, USA). To remove the attached antibody, the membrane was initially washed for 30 min with a 0.1 M (pH 3.0) glycine buffer, and then washed for 30 more minutes with 0.1 M Tris-HCl (pH 8.0) buffer. The blot was blocked with blocking buffer for 1 h 30 min, and then treated with ß-actin antibody to confirm equal protein loading.

### 2.4. Staining for Senescence Associated β-Galactosidase

SA-β-galactosidase staining was performed using a senescence detection kit (Cell Signaling Technology). Briefly, treated cells were stained with a staining solution at 37 °C for 24 h. As a result of microscopic observation, β-gal-positive stained cells were observed. The total number of cells and stained cells were counted to calculate the percentage of SA-β-gal-positive cells.

### 2.5. Detection of Superoxide and Nitric Oxide Formation

Cellular superoxide generation was detected using the fluorescent probe 2′,7′-Dichlorofluorescin diacetate (DCF-DA; Sigma-Aldrich, St. Louis, MO, USA) as previously described [13]. Treated HUVECs were treated with DCF-DA (10 μmol/L) for 30 min at cell culture temperature, then imaged using fluorescence microscopy. Next, to quantify reactive oxygen species (ROS) levels, HUVECs were seeded in 96 wells and treated as described, following which the fluorescence level was measured using fluorescence intensity of Ex/Em = 485/535 nm in a fluorescence reader (Versamax, Molecular Devices, Sunnyvale, CA, USA). Subsequently, NO production was measured using 4-Amino-5-Methylamino-2’,7’-Difluorofluorescein Diacetate (DAF-FM DA; Thermo Fisher Scientific, Waltham, MA, USA) at a concentration of 5 μM. After the washing process, the degree of NO production was quantified by completing the intracellular de-esterification reaction and measuring the fluorescence at Ex/Em 495/515 nm.

### 2.6. Statistical Analysis

All data are presented as means ± SD, and ANOVAs were used to examine for significant differences by comparing the means between two groups, followed by Tukey’s post hoc test and individual comparisons. Statistical significance was defined as a *p*-value < 0.05.

## 3. Results

### 3.1. Chrysanthemum coronarium L. (CC) Extract Reversed Senescence in HUVECs

**Figure 1:**
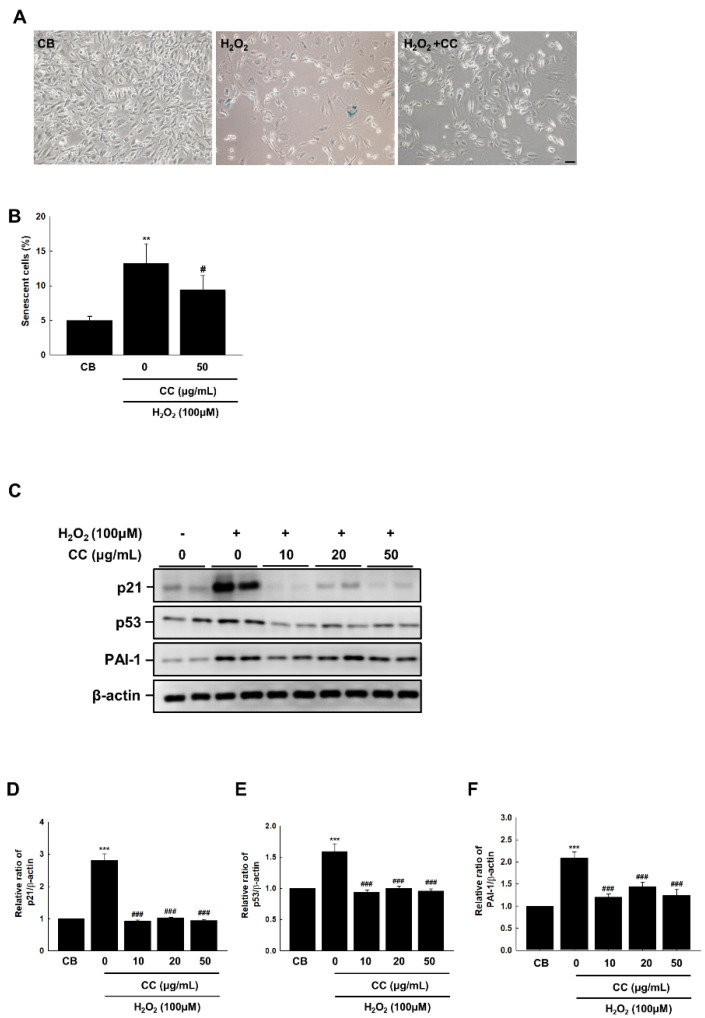
(**A**) *Chrysanthemum coronarium* L. (CC) extract reduces the senescent phenotype in H_2_O_2_ (100 μmol/L)-stimulated HUVECs, as shown by β-gal staining. The magnification was set to 40×. Scale bar = 50 μm. (**B**) Quantitative analysis of positive SA-β-gal stained cells. Data shown are derived from three independent experiments and expressed as the mean ± SEM (n = 6 per group). (**C**) CC reduced the expression of p21, p53, and PAI-1 induced by H_2_O_2_. (**D**–**F**) The relative ratios from comparison to β-actin in duplicate blots are shown in a graph chart. Control cells (CB) received the vehicle alone. The T Data shown are derived from three independent experiments and expressed as the mean ± SEM (*n* = 6 per group). **, *p* < 0.01, ***, *p* < 0.001, versus CB; #, *p* < 0.05, ###, *p* < 0.001, vs. H_2_O_2_.

To evaluate the effects of CC on endothelial premature senescence, we investigated H_2_O_2_-induced premature senescence in HUVECs via SA-β-gal staining. HUVECs treated with 100 μM H_2_O_2_ were stained with SA-β-galactosidase for 24 h. As shown in Figure 1A, senescent cells appeared blue and their number significantly decreased following CC treatment.

H_2_O_2_ increased the expression of p53 and p21, both known cell cycle controllers, as well as thrombosis and atherosclerosis markers and endothelial plasminogen activator inhibitor-1 (PAI-1), a regulator of aging-associated thrombosis [14] (Figure 1C). Conversely, in concentrations of 10 μM and greater, CC reduced the expression of p21 and p53, indicating endothelial senescence regulation.

### 3.2. Chrysanthemum coronarium L. (CC) Extract Reduces Oxidative Stress-Induced Endothelial Senescence in HUVECs

**Figure 2:**
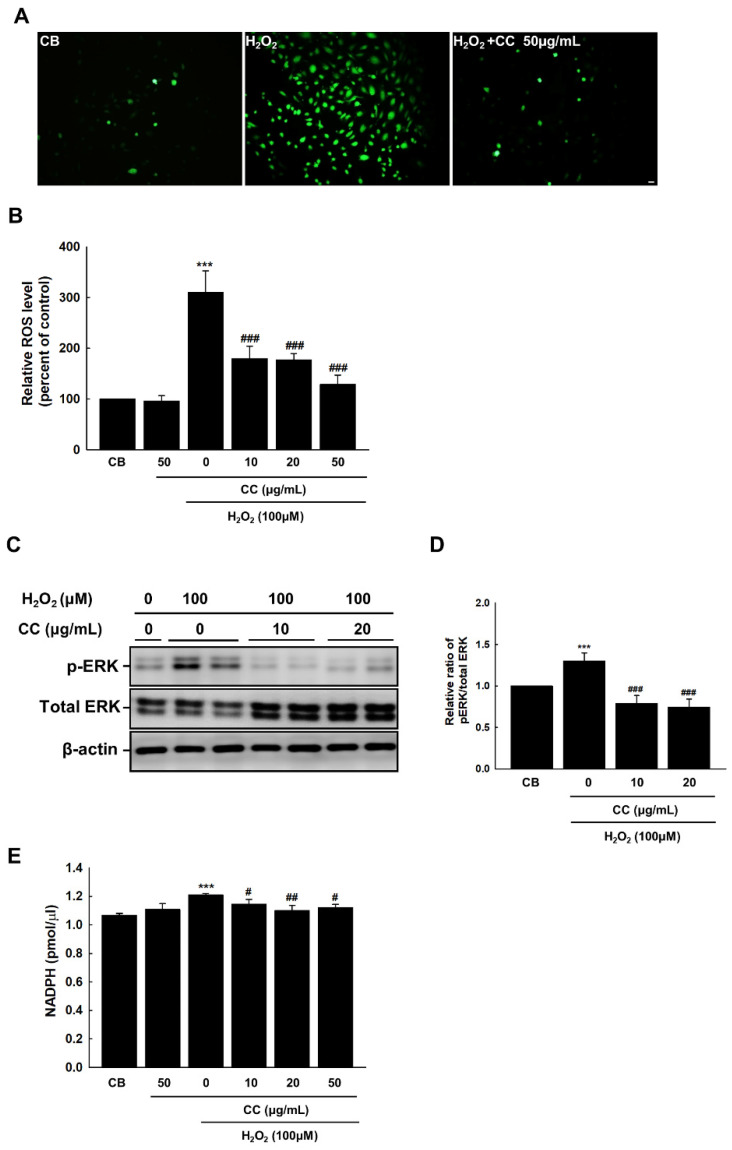
*Chrysanthemum coronarium* L. (CC) extract reduces endothelial senescence in HUVECs induced by ROS. (**A**) Fluorescent staining images illustrating ROS production in HUVECs via DCF-DA staining. The relative ratios of DCF-DA fluorescence intensities indicate the degree of endothelial ROS formation in HUVECs. The original magnification was set to 20 ×. Scale bar = 50 μm. (**B**) The fluorescence sensitivity of DCF-DA was standardized for the total number of cells in each dish. (**C**–**D**) Expression of phospho-ERK, total ERK, and β-actin in H_2_O_2_-treated cells. (**E**) NADPH was measured using a colorimetric assay. Control cells (CB) received the vehicle alone. Bars represent the mean ± SEM from three dishes per group. ***, *p* < 0.001, versus CB; #, *p* < 0.05, ##, *p* < 0.01, ###, *p* < 0.001, vs. H_2_O_2_.

Elevated ROS levels lead to oxidative stress, which is associated with a myriad of inflammatory and degenerative pathologies. In ECs, ROS interferes with vascular growth and has a profound effect on the regulation of cell death. To examine the effect of CC extract on the reactive oxidative stress response of H_2_O_2_-stimulated ECs, intracellular ROS levels were measured through treatment with Fluorescent-based DCF-DA. CC extract dramatically reduced intracellular ROS levels in senescent ECs in a dose-dependent manner (Figure 2A,B). Mitogen-activated protein kinases, which contain extracellular signal-regulated kinase (ERK), are involved in regulating cellular communication related to cell growth, death, and survival [15]. We found that treatment with CC extract reduced ERK phosphorylation in ECs exposed to oxidative stress (Figure 2C,D). To determine the effect of CC extract on NADPH, we measured the amount of accumulated ROS due to NADPH oxidase activation. NADPH levels were measured via H_2_O_2_ stimulation, and CC extract reduced NADPH concentration at concentrations of 10, 20, and 50 μg/mL (Figure 2E).

### 3.3. Chrysanthemum coronarium L. (CC) Extract Reduces Amount of NO Generated from Endothelium to Prevent Senescence in HUVECs

**Figure 3:**
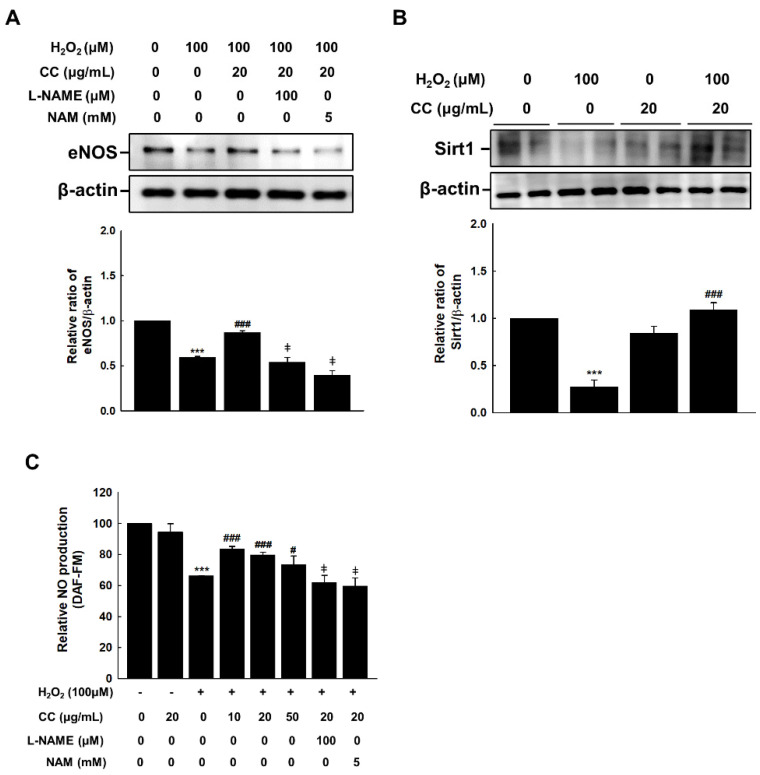
*Chrysanthemum coronarium* L. (CC) extract improves endothelial NO formation to combat senescence in HUVECs. (**A**) CC reversed the H_2_O_2_ (100 μmol/L)-induced decrease of eNOS expression in H_2_O_2_-, L-NAME- (100 μM), and NAM- (5 mM) treated HUVECs. (**B**) Expression of Sirt1 in H_2_O_2_ and H_2_O_2_ + CC HUVECs (**C**) The relative ratios of DAF-FM fluorescence intensities indicate the degrees of endothelial NO formation in the H_2_O_2_-, L-NAME- (100 μM), and NAM- (5 mM) treated HUVECs. ***, *p* < 0.001, versus CB; #, *p* < 0.05, ###, *p* < 0.001, versus H_2_O_2_; ‡, *p* < 0.05, vs. H_2_O_2_ + CC.

eNOS is an enzyme that synthesizes endothelial NO. To investigate the effect of CC extract on this enzyme, we treated H_2_O_2_-stimulated HUVECs with anti-eNOS and anti-Sirt1 antibodies. Treatment with CC extract recovered the eNOS and Sirt1 expression in HUVECs that had been inhibited by H_2_O_2_ treatment (Figure 3A,B). While CC extract restored the reduction in eNOS and NO production induced by H_2_O_2_, this recovery effect was blocked by the eNOS inhibitor Nω-nitro-l-arginine methyl ester (L-NAME) and Sirt1 inhibitor nicotinamide (NAM) (Figure 3C).

Disruption of NO formation is a sign of endothelial senescence. In the present study, a DAF-FM probe was used to detect NO formation in endothelial cells. As shown in Figure 3C, the fluorescence intensity of NO decreased following H_2_O_2_ treatment, and this effect was reversed by CC treatment in a dose-dependent manner.

### 3.4. UPLC-Q-TOF Mass Analysis

**Figure 4:**
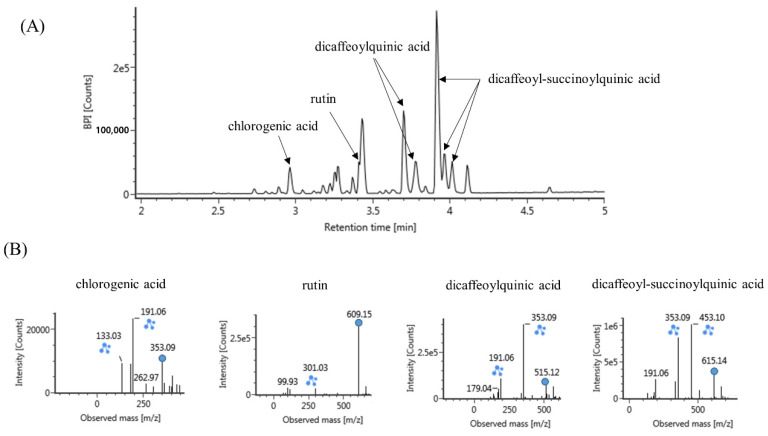
(**A**) Representative chromatogram of *Chrysanthemum coronarium* L. extract (**B**) and spectra of major compounds. The extract was analyzed using UPLC-Q-TOF MS and its major compounds were tentatively identified using the online databases connected to the UNIFI software (Waters).

To analyze the compounds present in the CC extract, we used UPLC-Q-TOF mass spectrometry. The results indicated the presence of chlorogenic acid, rutin, dicaffeoylquinic acid, and dicaffeoyl-succinoylquinic acid in CC extract (Figure 4).

## 4. Discussion

Senescence is a stage in which the cell cycle is irreversibly terminated, and division stops in response to oxidative stress and various other stresses in the cell [16]. In this study, we established a model of H_2_O_2_-induced senescence using HUVECs to examine the protective effect of CC extracts against cellular senescence. The results showed that, when treated with 100 μM H_2_O_2,_ the number of SA-β-gal-positive cells increased by about three times compared with the control, whereas this number decreased following treatment with CC extract, demonstrating the efficacy of CC in preventing senescence in HUVECs. Thus, our results demonstrated the protective potential of CC extracts in cellular aging and elucidated this mechanism of action.

p21 expression signals that senescence has begun. Based on clinical findings, aging has been shown to be a strong inducer of p21 expression. Comparisons of ECs obtained from the antecubital veins and brachial arteries in young and elderly individuals found that aging was associated with 23% and 120% increased p21 expression in arteries and veins, respectively [17]. In this study, we confirmed that the CC extract can ameliorate endothelial senescence by regulating p21 and p53 expression; however, there was no change in p16 expression (results not shown) in H_2_O_2_-stimulated endothelial cells. The p53/p21cip1 and/or p16INK4A pathways are the best-known pathways for regulating cellular senescence [18]. Our results indicate that, through the various stress factors that trigger senescence CC extract, may primarily activate the p53 pathway as opposed to the p16INK4A pathway.

As an eNOS inhibitor, L-NAME inhibits the anti-aging effects of NO and induces endothelial and vascular aging [7]. In this study, we used a fluorescence-based DAF-FM fluorescent probe to detect decreased NO production following H_2_O_2_ treatment. After treatment with CC extract, NO production increased, suggesting that CC may exert an effect on NO production. Additionally, L-NAME inhibited the recovery effect associated with CC extract treatment on decreased NO production and anti-aging endothelium, further supporting the conclusion that the anti-aging effect of CC is a result of improved NO production.

SIRT1 is expressed in vascular ECs and is known to play a critical role in endothelial function [19]. In aging HUVECs, SIRT1 levels gradually decrease [20]. Therefore, researchers have studied SIRT1/eNOS as potential targets for regulating the mechanisms through which vascular dysfunction occurs during aging. SIRT1 acetylates eNOS, affecting its activity and NO formation. Under oxidative conditions, eNOS acetylation can induce endothelial dysfunction [21]. Therefore, our results suggest that CC extract treatment can effectively delay HUVEC aging through the regulation of eNOS and SIRT1.

In this study, the probable bioactive compounds responsible for CC extract’s anti-senescence effect on HUVECs can be predicted from various published reports. Most of these compounds show natural antioxidant effects, such as α-tocopherol, chlorogenic acid, luteolin, and rutin [22,23]. Furthermore, it has been reported that chlorogenic acid reduces angiotensin II-induced vascular senescence through the Nrf2/HO-1 pathway in vitro and in vivo [24]. Caffeoylquinic acid, another component of CC extract, is a natural polyphenol, and has been shown to exert antioxidant and neuroprotective effects, as well as prevent spatial learning and memory deficits in aging-accelerated mice models of Alzheimer’s disease [25]. Therefore, the antioxidant and anti-vascular aging effects of CC extract are assumed to be due to chlorogenic acid and other active components.

## 5. Conclusions

In this study, we showed that CC extract inhibited oxidative stress-induced premature senescence by regulating p21, p53, and PAI-1 expression. Furthermore, the increased expression of SIRT1 induced by CC extract played a critical role in preventing endothelial senescence by regulating eNOS expression, ultimately resulting in increased endothelial NO production. These results provide an important new perspective on the effects of CC extract against cellular aging and cardiovascular dysfunction.

## Data Availability

All the data analyzed for this manuscript are included. The analyzed raw data are available upon reasonable request to the corresponding author.
